# Antifungal and Antibiofilm Activities of B-Type Oligomeric Procyanidins From *Commiphora leptophloeos* Used Alone or in Combination With Fluconazole Against *Candida* spp.

**DOI:** 10.3389/fmicb.2021.613155

**Published:** 2021-02-22

**Authors:** Renato Dantas-Medeiros, Ana Caroline Zanatta, Luanda Bárbara Ferreira Canário de Souza, Júlia Morais Fernandes, Bruno Amorim-Carmo, Manoela Torres-Rêgo, Matheus de Freitas Fernandes-Pedrosa, Wagner Vilegas, Thiago Antǒnio de Sousa Araújo, Sylvie Michel, Raphaël Grougnet, Guilherme Maranhão Chaves, Silvana Maria Zucolotto

**Affiliations:** ^1^Laboratory of Pharmacognosy, Department of Pharmaceutical Sciences, Faculty of Pharmacy, Federal University of Rio Grande do Norte, Natal, Brazil; ^2^Laboratory of Bioprospecting of Natural Products, São Paulo State University (UNESP), São Paulo, Brazil; ^3^Laboratory of Phytochemistry, Institute of Chemistry, São Paulo State University (UNESP), São Paulo, Brazil; ^4^Laboratory of Medical and Molecular Mycology, Department of Clinical and Toxicological Analyses, Federal University of Rio Grande do Norte, Natal, Brazil; ^5^Laboratory of Technology and Pharmaceutical Biotechnology (Tecbiofar), Faculty of Pharmacy, Federal University of Rio Grande do Norte, Natal, Brazil; ^6^Department of Health, University Center of Maurício de Nassau, Recife, Brazil; ^7^Laboratory of Pharmacognosy, Faculty of Pharmacy, University Paris Descartes, Paris, France

**Keywords:** Burseraceae, fungal infections, biofilm, resistance, herbal drug, natural antifungal, imburana

## Abstract

*Commiphora leptophloeos* (Burseraceae) is a medicinal plant native to Brazil which is popularly used for treating oral and vaginal infections. There has been no scientific evidence pointing to its efficacy in the treatment of these infections. Thus, this study sought to investigate the cytotoxic, antifungal, and antibiofilm activity of *C. leptophloeos* against *Candida* spp. and to isolate, identify, and quantify the content of B-type oligomeric procyanidins (BDP) in the extract of *C. leptophloeos* stem bark. The extract and the *n*-butanol fraction were obtained by maceration and liquid-liquid partition, respectively. Phytochemical analysis performed by HPLC-PDA/ELSD and FIA-ESI-IT-MS/MS allowed the identification and quantification of BDP in the samples. The application of centrifugal partition chromatography helped isolate BDP, which was identified by ^1^H NMR and MS analyses. *Candida* spp. reference strains and clinical isolates (including fluconazole-resistant strains) derived from the blood cultures of candidemic patients and the vaginal secretion of patients with vulvovaginal candidiasis were used for evaluating the antifungal and antibiofilm effects. Minimal inhibitory concentration (MIC) and minimal fungicidal concentration (MFC) were determined by the microdilution technique, and biofilm inhibition was evaluated through crystal violet and XTT assays. The combined action of BDP with fluconazole was determined by the checkerboard method. The extract, the *n*-butanol fraction, and the BDP exhibited antifungal activity with MIC values ranging from 312.5 to 2500 μg/mL and were found to significantly reduce the biofilm formed in all the *Candida* strains investigated. BDP showed a fungicidal potential against strains of *Candida* spp. (especially against fluconazole-resistant strains), with MIC and MFC values ranging from 156.2 to 2500 μg/mL. In addition, the combined application of BDP and fluconazole produced synergistic antifungal effects against resistant *Candida* spp. (FICI = 0.31–1.5). The cytotoxic properties of the samples evaluated in human erythrocytes through hemolytic test did not show hemolytic activity under active concentrations. The findings of the study show that *C. leptophloeos* has antifungal and antibiofilm potential but does not cause toxicity in human erythrocytes. Finally, BDP, which was isolated for the first time in *C. leptophloeos*, was found to exhibit antifungal effect against *Candida* spp. either when applied alone or in combination with fluconazole.

## Introduction

Several microorganisms are part of the normal human microbiota, and these include the fungi from the *Candida* genus. Sixty five percent of *Candida* spp. are able to grow at temperatures below 37°C; this facilitates the maintenance of their commensal state in the human host (Azzam et al., 2020). However, complex changes in microbial communities may cause various disorders, such as opportunistic fungal infections (Zelante et al., 2020). Candidiasis is the name given to a broad range of infections caused by the *Candida* species (Bonifácio et al., 2019). It is worth noting that *Candida* spp. have been found to be responsible for several fungal infections, such as oral and vulvovaginal candidiasis, skin infections, and onychomycosis; these infections may occur even in immunocompetent hosts, when predisposing factors are present (Fakhim et al., 2020).

Candidemia is a bloodstream infection caused by *Candida* spp.; this infection has been found to have a high mortality rate (35–75%) which varies between regions and countries (Kaur and Chakrabarti, 2017; Fakhim et al., 2020). Among the *Candida* species, *Candida albicans* has been found to be the most prevalent species, responsible for about 80–90% of the cases involving Candidemia (Robbins et al., 2017). Although *C. albicans* is the most virulent and isolated species from the bloodstream of hospitalized patients, in recent years, studies published have pointed to a noticeable increase in the occurrence of infections caused by clinically relevant non-*C. albicans Candida* (NCAC) species, such as *Candida tropicalis*, *Candida dubliniensis*, *Candida krusei*, *Candida glabrata*, and *Candida parapsilosis* (Biswal et al., 2017; Banerjee et al., 2019).

The ability to form biofilms is one of the main virulence factors of *Candida* spp., particularly in *C. albicans* (Deorukhkar and Saini, 2016). Biofilms are communities of microorganisms attached to biotic or abiotic surfaces and which are surrounded by a matrix of polymeric extracellular substances that are difficult to eliminate (Sherry et al., 2017). The matrix protects fungal cells, preventing the penetration of antifungal drugs and the attack of phagocytic cells; this impairs the successful treatment of candidemia, specifically in those patients harboring a central venous catheter (Souza et al., 2018).

Currently, the main antifungal drugs commercially available are azoles, polyenes, echinocandins, and nucleoside analogs. Although there appears to be a reasonable number of antifungal drugs in the market, very few of these drugs, including amphotericin B and echinocandins, are effective against fungal infections associated with biofilm formation (Sherry et al., 2017; De Cássia Orlandi Sardi et al., 2018). In addition, these antifungal drugs have been found to have some limitations, which include increased antifungal resistance (mainly due to emerging *Candida* species), high costs, and high degree of toxicity in humans (Colombo et al., 2017; Spivak and Hanson, 2018). The emergence of multidrug-resistant fungi (for instance, *Candida auris*) makes the available therapeutic arsenal for the treatment of the infection even more limited (Nett, 2019). Other *Candida* species, such as *Candida glabrata, Candida krusei, Candida lusitaniae, Candida kefyr, Yarrowia (Candida) lypolitica, and Candida rugose*, have also been found to exhibit multidrug resistance (Colombo et al., 2017). New treatments which involve the combined application of antifungal drugs with synergistic effects have been shown to be a promising alternative to overcome the fungal resistance to current antifungal drugs (Fakhim et al., 2017). The interaction between micafungin and voriconazole has shown synergistic activity against multidrug-resistant *C. auris* (Fakhim et al., 2017). Also, the combined use of fluconazole and plant extracts has been found to enhance the ability of fluconazole to inhibit the expression of virulence factors of *Candida* spp. (Morais-Braga et al., 2016).

In this context, the discovery of new therapeutic agents that are highly efficient for the treatment of emerging opportunistic fungal infections is, undoubtedly, of great clinical relevance (Campoy and Adrio, 2017; Diba et al., 2018). The urgent need for the search of new antifungal drugs provides an impetus for the research and exploration of novel therapeutic alternatives from different natural sources (Sardi et al., 2013). Medicinal plants for the treatment of fungal infections are found to be highly promising in the sense that they have significant antimicrobial activity (either acting alone or in combination with antifungal drugs) and low degrees of toxicity, in addition to being less costly (Silva-Rocha et al., 2017).

*Commiphora leptophloeos* (Mart.) J.B. Gillett (Burseraceae) species is a medicinal plant native to the caatinga biome of the northeastern region of Brazil; the plant species is popularly known as “imburana” or “imburana-de-cambão.” The stem bark of *C. leptophloeos* is widely used in traditional medicine for topical treatment of oral and vaginal infections, wounds, and inflammation-related problems (Agra et al., 2007; Cartaxo et al., 2010; Macedo et al., 2018). To date, few studies published in the literature have investigated the phytochemical and pharmacological properties of the stem bark of *C. leptophloeos*. Most non-clinical studies published in the literature have been mainly confined to investigating the antibacterial and antibiofilm activities, as well as the chemical composition of *C. leptophloeos*; these studies have reported the presence of phenolic acids, *O*- and *C*-glycosylated flavonoids, lignans, and A- and B-type polymeric proanthocyanidins in *C. leptophloeos*, where the identified compounds have been related to therapeutic activities of the plant species (Trentin et al., 2013; Pereira et al., 2017; Da Silva et al., 2019; Dantas-Medeiros et al., 2021).

Considering the importance of *C. leptophloeos* in traditional medicine, coupled with the lack of scientific studies on this plant species, the present work sought to investigate the cytotoxicity and antifungal and antibiofilm activities of B-type oligomeric procyanidins from *Commiphora leptophloeos* alone or in combination with fluconazole against *Candida* spp. The study also aimed to isolate, characterize, and quantify the active compounds present in the stem bark of *C. leptophloeos*.

## Materials and Methods

### Chemicals and Reagents

HPLC-grade acetonitrile and formic acid were acquired from J. T. Baker (Brazil) and LC-MS grade methanol (from Chromasolv) was used as mobile-phase components in the chromatographic analysis. Deuterated solvents were acquired from Euriso-Top (Saint Aubin, France). Folin-Ciocalteu reagent, Triton X-100, Yeast Nitrogen Base medium (YNB), D-glucose monohydrated, XTT/menadione, and fluconazole were purchased from Sigma-Aldrich (St. Louis, Missouri, United States of America). Mueller-Hinton medium was acquired from HiMedia (Mumbai, Maharashtra, India). Sabouraud Dextrose Agar (SDA) was acquired from Basingstoke, Hampshire, United Kingdom. RPMI 1640 culture medium (Roswell Park Memorial Institute) was acquired from Angus buffers and Biochemical, Niagara Falls, New York, United States of America. All other reagents and solvents used in the experiments were of analytical grade. The water used was purified by Milli-Q reverse osmosis (Millipore).

### Plant Material

*Commiphora leptophloeos* stem barks were collected in the community of Carão, Altinho (8° 29′ 32” latitude and 36° 03′ 03” longitude), Pernambuco, in the northeastern region of Brazil, in July 2016. The vegetal material was identified in the Geraldo Mariz Herbarium of the Federal University of Pernambuco (UFPE) (number 46.191). Permission to collect the material was issued by the Brazilian Authorization and Biodiversity Information System (SISBIO) (process number 35017). The National System for the Management of Genetic Heritage and Associated Traditional Knowledge (SISGEN) gave the authorization for the conduct of the scientific research (process number A618873).

### Preparation of Hydroethanolic Extract and Procyanidins-Rich Fraction

The stem bark was dried in a circulating air oven (temperature below 45°C), and then ground in a knife mill. An amount of 200 g of the powder material was extracted with ethanol: water (70:30, v/v) by maceration for 48 h in a plant/solvent proportion of 1:10 (w/v) (extract 1). The extract was filtered through Whatman^TM^ paper number 1 and the same vegetal material was extracted (remaceration) under the same conditions previously described (extract 2). Both extracts were combined and concentrated under reduced pressure using a rotary evaporator (temperature below 35°C) (Buchi-Model V-700, Altendorfer Str. 3, Essen, Germany) in order to remove the organic solvent; this yielded the hydroethanolic extract of *Commiphora leptophloeos* (named HECL). The extract was frozen and lyophilized at 200 mT for 72 h at −60°C (Model 101, Liotop^®^, São Carlos, São Paulo, Brazil).

The procyanidins-rich fraction was obtained based on the technique proposed by Rohr et al. (2000) with some modifications. In brief, 100 g of the HECL was subjected to liquid–liquid extraction with solvents of increasing polarity (dichloromethane, ethyl acetate, and *n*-butanol) and water, in the ratio of 2:1 (extract: solvent, v/v), to obtain the oligomeric procyanidins-rich *n*-butanolic fraction (BF). For each solvent, three partitions were made, each one starting from 200 of extract to 100 mL of organic solvent (3 × 100 mL). The BF was concentrated using a rotary evaporator (temperature below 35°**C**); the fraction was then lyophilized and stored at −20°C. The HECL and BF amounted to 25.6 and 12.5 g, respectively.

### Pretreatment of Sample

The HECL and BF were treated using solid phase extraction (SPE; Chromabond^®^, 45 μm, 500 mg, 6 mL). The cartridges were pre-activated with MeOH (3.0 mL) and equilibrated with H_2_O (3.0 mL). An amount of 50 mg of the dry extract was dissolved in EtOH/H_2_O (85:15 v/v), loaded to a C-18 cartridge, and eluted sequentially with 3 mL of the same mobile phase. The solvent was dried under N_2_ atmosphere.

### Phytochemical Characterization by Mass Experiments

The samples were separately analyzed through direct injection by flow injection-electrospray ionization-ion trap-tandem mass spectrometry (FIA-ESI-IT-MS/MS) technique. All the samples were diluted in pure methanol to a concentration of 10 ppm for HECL and BF, and 2 ppm for BDP. The MS analysis was conducted using a Thermo Scientific LTQ XL linear ion trap mass spectrometer (acquired from Thermo–San Jose, California, United States of America) equipped with an electrospray ionization (ESI) source in negative mode. The following was applied for the analysis: a fused-silica capillary tube at 280°C, with spray voltage of 5.00 kV, capillary voltage of −47 V, tube lens of −226 V, and flow rate of 5.0 μL/min. The working range applied for the full scan analysis varied from *m/z* 150–1,500. The systems were pre-selected through a full scanning of the MS to acquire data related to the ions within the established *m/z* range. Thereafter, MS/MS experiments were performed based on the data for the precursor ions of interest with 30% of collision energy and an activation time of 30 ms. The data were acquired and processed using the Xcalibur^TM^ version 1.3 software (from Thermo Finigan, San Jose, California, United States of America).

### Centrifugal Partition Chromatography (CPC) Purification Procedure for Isolation of B-Type Dimeric Procyanidin (BDP)

#### Distribution Coefficient (K) Estimation and Solvents System Selection

The solvents system was selected based on an estimation of the distribution coefficient (K), defined as the ratio of concentrations of the targeted compounds between the two non-miscible phases (Hopmann et al., 2012). K was evaluated by the shake-flask method and thin layer chromatography (TLC) under the following conditions: small amounts (about 2 mg) of the BF were added to the previously equilibrated biphasic solvents systems in test vials and were vigorously shaken for 2 min. After decantation, TLC analysis was performed under ultraviolet-visible light (UV) and sprayed with a solution of sulfuric vanillin; the mobile phase system obtained comprised the following: AcOEt/MeOH/C_3_H_6_O/H_2_O (0.1:1.8:1.1:0.5 v/v/v/v).

#### Isolation of B-Type Dimeric Procyanidin

The BF was subjected to preparative analysis by CPC. The CPC analysis was performed using a double rotor CPC- 250 + 1,000-B apparatus (Gilson Purification SAS, Saint-Avé, France) with an inbuilt quaternary high-pressure gradient pump and 1,953 ovoid twin-cell technology cells for the 250 mL column. The AcOEt/MeOH/C_3_H_6_O/H_2_O (0.1:1.8:1.1:0.5 v/v/v/v) system was prepared in a separation funnel, vigorously shaken, and left idle at room temperature (25°C) until the separation of the phases. CPC was operated in ascending mode at room temperature with rotational speed set at 1,200 rpm. The mobile phase was pumped through the column at the desired flow rate of 20 mL/min.

A sample containing 6.0 g of BF was dissolved in 45 mL of biphasic solvents system (35 mL of upper phase and 15 mL of lower phase) and injected through a 50 mL loop. This experiment yielded 25 sub-fractions (SF) which were evaluated by TLC using the same mobile phase described above. Finally, an analysis was conducted aimed at verifying whether the SF-16 was a pure compound; the analysis was carried out using proton nuclear magnetic resonance (^1^H NMR) and mass spectrometry (MS). After the analysis, the compound was identified as B-type dimeric procyanidin (named BDP).

### Nuclear Magnetic Resonance Analysis

The ^1^H NMR spectra of the isolated compound were recorded at 400 MHz with the aid of a Bruker Avance III 400 spectrometer, using Bruker pulse programs. NMR Fourier transform, integration, and peak picking analyses were conducted using Bruker TopSpin software version 3.2. Chemical shifts were reported in ppm and coupling constants (*J*) in Hertz. The isolated compound was dissolved in suitable deuterated methanol.

### Quantification of B-Type Oligomeric Procyanidins by Liquid Chromatography Analyses

To conduct the high-performance liquid chromatography-photodiode array/evaporative light scattering detection (HPLC-PDA/ELSD) analyses, the following equipment was employed: JASCO^®^ model ELS-2040/2041 HPLC and JASCO^®^ automatic pump model of quaternary pump, coupled to photodiode detection (PDA) and evaporative light scattering detector (ELSD). LC separations were performed using a C-18 column (Thermo Scientific^®^ RO, 250 × 4.6 mm, 5 μm particle size). The mobile phase—which was composed of water (solvent A) and acetonitrile (solvent B), both acidified with 0.1% formic acid–was used for the samples elution. The optimized gradient method was employed: 10–20% B, 0–7 min; 20% B, 7–25 min, and 20–60% B, 25–45 min. The flow rate of 0.8 mL/min and injection volume of 10 μL were applied. The UV-PDA detector was programmed at the wavelength of 150–550 nm and the chromatograms were plotted at 280 nm. The ELSD was operated with a drift tube temperature of 40°C and nebulizer gas pressure of 3.5 bar. The HECL and BDP dried samples were re-suspended in pure MeOH in a concentration of 10 and 3 mg/mL, respectively, and filtered through a Millex^®^ PTFE filter (0.22 μm, 25 mm). The analysis of each sample was performed in triplicate. Chromatographic data acquisition and processing were performed using the ChromNAV software (Chromatec^®^).

The quantification technique applied here was conducted in line with the Brazilian legislation no. 166, promulgated on July 24, 2017 (Anvisa, 2017) and under the regulatory guidelines Q2 (R1) of the International Conference on Harmonization of Technical Requirements for Registration of Pharmaceuticals for Human Use (ICH) (Singh, 2015). For the quantification analysis, the following parameters were evaluated: specificity, selectivity, calibration curve, linearity, limit of detection (LOD), limit of quantification (LOQ), and precision. The specificity of the method was conducted by co-injection of the BDP (purity > 95% HPLC) and the extract in order to evaluate the increase in peak area. The index of similarity between the UV spectrum of the standard (BDP) and the UV spectrum of each peak in the chromatographic profile was calculated in order to verify the different compounds with similar UV absorption to the BDP in the extract (HECL). Finally, the HPLC-ELSD analysis was conducted in order to quantify all the peaks observed in the extract. The selectivity of the method was determined by analyzing and comparing the chromatographic data (UV spectrum, retention time, and peak spectral purity) of HECL with the data obtained for the BDP in triplicate.

The BDP analytical curve was constructed using five concentration levels (250, 500, 1,000, 1,250, and 1,500 μg/mL). Each concentration level was analyzed in triplicate. The average of the chromatographic peak areas in the ELSD chromatogram was interpolated as a function of known concentrations of BDP; this was done in order to establish the linear regression equation and the correlation coefficients. The limit of detection (LOD) and the limit of quantification (LOQ) were determined based on signal-to-noise ratios of 3.3:1 and 10:1, respectively. The external standard method was applied in order to quantify each peak of the extract. The peaks 1–5 of the extract were quantified using the BDP calibration curve.

### Determination of Total Polyphenolic Content (TPC)

The total polyphenol content (TPC) of HECL and BF was estimated based on the Folin-Ciocalteu (F-C) reagent method, as previously reported, with slight modifications (Attard, 2013). In a 96-well plate, an amount of 25 μL of sample solution at 2 mg/mL was mixed with 125 μL of F-C reagent freshly diluted to 1:10 (v/v) with distilled H_2_O. After 5 min, an amount of 100 μL of the solution was mixed with 100 μL of a 7.5% Na_2_CO_3_ solution; the mixture was then left for 30 min in the dark and the absorbance was measured in a microplate reader (Epoch-Biotek^®^, Winooski, Vermont, United States of America) at 765 nm. The respective standard curves of gallic acid (1.25, 2.5, 5, 10, 20, 40, 60, and 80 μg/mL) and the blank (replacing F-C reagent with 125 μL H_2_O) were constructed simultaneously. Thereafter, the total phenol content was calculated as mg of gallic acid (EAG) equivalent per g of the sample (mg of EAG/g of sample). For each sample, the analysis was performed in triplicate (*n* = 3).

### Toxicity Study

#### *In vitro* Hemolytic Activity Assay

The toxicity of HECL, BF, and BDP was evaluated by hemolytic assay using healthy human donor blood, after obtaining written informed consent and approval from the Research Ethics Committee of the Onofre Lopes University Hospital–HUOL/UFRN/Brazil (process number 3.676.695). The assay employed was based on the methodology described by Amorim-Carmo et al. (2019), where an erythrocyte suspension (2% v/v) was washed 3 times in 0.9% saline (pH 7.4) and centrifuged at 1,500 rpm for 10 min. Human erythrocytes were incubated for 1 h at 37°C with the HECL, BF, or BDP at different concentrations (19.5–625 μg/mL) and centrifuged at 1,500 rpm for 10 min. The content of hemoglobin present in the supernatant was quantified at 540 nm using a microplate reader. The positive control of the assay was 1% (v/v) Triton X-100 solution (100% cell lysis) and the negative control applied was 0.9% saline solution (0% cell lysis). The values were expressed as mean ± S.D. (*n* = 3).

### Antifungal and Antibiofilm Activities

#### Selection of Microorganisms and Culture Conditions

In order to investigate the antifungal, antibiofilm, and synergistic effects of the extract, nine clinical isolates of *Candida* spp. were obtained from the blood cultures of patients with candidemia, as well as from vaginal secretion of patients with vulvovaginal candidiasis. The research project was approved by the Local Research Ethics Committee (“Comitê de Ética em Pesquisa da Liga Norte Riograndense Contra o Câncer”), under the protocol number 042/042/2012. Written consent from the patients was not required because of the observational nature of the study, coupled with the fact that no clinical conditions and/or demographic data of patients are displayed in the manuscript.

The bloodstream isolates, namely, *C. tropicalis* LMMM195, *C. tropicalis* LMMM447, *C. krusei* LMMM249, *C. glabrata* LMMM704, *C. parapsilosis* LMMM83, and *C. parapsilosis* LMMM85, along with *C. albicans* strains (LMMM74, LMMM92, and LMMM100) from vaginal secretion were used in the experiments. In addition, reference strains, including *C. albicans* ATCC 90028, *C. tropicalis* ATCC 13803, *C. parapsilosis* ATCC 22019, *C. glabrata* ATCC 2001, and *C. krusei* ATCC 6258, provided by the American Type Culture Collection (ATCC) and the Netherlands Collection Centraalbureau voor Schimmelcultures (CBS), were also employed in the study. The strains were stored at −80°C in Yeast Extract-Peptone-Dextrose (10 g/L yeast extract, 20 g/L glucose, and 20 g/L mycological peptone) containing 20% (v/v) glycerol. All the strains belong to the mycological culture collection of the Laboratory of Medical and Molecular Mycology, Department of Clinical and Toxicological Analyses, Federal University of Rio Grande do Norte, Brazil.

#### Determination of Minimal Inhibitory Concentration (MIC)

The antifungal susceptibility test was performed using the broth microdilution assay based on protocol M27-A3 of the Clinical and Laboratory Standards Institute (CLSI, 2008), for defining the minimal inhibitory concentrations (MICs) of fluconazole, HECL, BF, and BDP. Fluconazole solution was prepared in line with M27-A3 guidelines; the solution was diluted in RPMI 1640 buffered with 3-(N-morpholino) propanesulfonic acid (MOPS) at pH 7.0. All the natural products tested were re-suspended in the same culture medium recommended by the CLSI, after solubilization in 0.5% dimethyl sufoxide (DMSO). To standardize the inoculum, *Candida* spp. cells were initially cultivated for 24 h using Sabouraud Dextrose Agar (SDA) at 35°C, and an initial suspension was adjusted spectrophotometrically to match the 0.5 McFarland scale standard (1–5 × 10^6^ cells) at 530 nm. Subsequently, two serial dilutions were made, the first in saline solution (1:100), and the second in RPMI 1640 (1:20), in order to obtain the final concentration of 10^3^ cells/mL. Afterward, aliquots of 100 μL of the final inoculum solution were added to each well with 100 μL of different concentrations (2,500–0.001 μg/mL) of HECL, BF, or BDP and (64–0.03 μg/mL) fluconazole. Finally, the microtiter plates were incubated at 37°C and the reading was done after 24 h of incubation for fluconazole, and between 24 and 48 h for the natural products. In addition, the reference strains *C. parapsilosis* ATCC22019 (susceptible) and *C. krusei* ATCC6258 (resistant) were included as control microorganisms for the testing of fluconazole. The MIC was defined as the lowest concentration which showed about 50% reduction in visual growth as compared to the positive control for fluconazole, HECL, BF, or BDP. The isolates were classified as resistant at the following cutoff point: MIC ≥ 8 μg/mL to fluconazole for all the *Candida* spp. tested, except for *C. glabrata*, where strains with MIC ≥ 64 μg/mL were considered resistant. *Candida krusei* is intrinsically resistant to fluconazole (CLSI, 2017).

#### Determination of Minimal Fungicidal Concentration (MFC)

In order to confirm whether the contractions of HECL, BF, and BDP which visually inhibited 100% of yeast cells’ growth in the wells corresponded to the MFC, 10 μL aliquots of each well, where no visual growth was detected, and using the same conditions for both the positive and negative controls, were seeded on the surface of SDA and the plates were incubated for 48 h at 37°C. The MFC was defined as the lowest concentration of natural products which resulted in no colony forming unit (CFU) growth.

#### Biofilm Assay

Biofilm assays were performed based on the method proposed by Chaves et al. (2013). First, 100 mL aliquots of a standardized cell suspension (10^7^ cells/mL) were transferred to microtiter plates and incubated for 1.5 h at 37°C in a shaker at 75 rpm. Following the adhesion phase, cell suspensions were aspirated and each well was washed twice with PBS to remove loosely adherent cells. A total of 100 μL of Yeast Nitrogen Base medium (YNB) with 50 mM of g D-glucose monohydrate containing 1,250 μg/mL of HECL and 625 μg/mL of BF and BDP were added to each well and incubated at 37°C in a rotatory incubator (Tecnal, TE-420, São Paulo, Brazil) at 75 rpm. Biofilms were allowed to develop for 72 h and were then quantified by crystal violet staining and XTT-reduction assays [2,3-bis(2-methoxy-4-nitro-5-sulfophenyl)-2*H*-tetrazolium-5-carboxanilide]. To perform the crystal violet assay, the biofilm-coated wells from the microtiter plates were washed twice with 200 μL of phosphate-buffered saline (PBS) and air dried for 45 min. Subsequently, each well was stained with 110 μL of 0.4% aqueous crystal violet solution for 45 min. Each well was then washed thrice with 300 μL of sterile distilled water and immediately distained with 200 μL of 95% ethanol for 45 min. Thereafter, an amount of 100 μL of the distaining solution was transferred to a clean well and the absorbance was measured with a microtiter plate reader at 570 nm. For the XTT-reduction assay, the biofilm-coated wells of the microtiter plates were washed twice with 150 μL of PBS and an amount of 100 μL of XTT/menadione solution (1 μM of menadione) was added to each well containing a prewashed biofilm and the negative control wells. The plates were covered with aluminum foil and incubated in the dark for 2–3 h at 37°C. Subsequently, an amount of 75–80 mL of the solution was removed (the resulting colored supernatant from each well) and transferred to the wells of a new microtiter plate. The solution was measured with a microtiter plate reader at 490 nm.

#### Analysis of the Synergistic Action of B-Type Dimeric Procyanidin (BDP) From *Commiphora leptophloeos* With Fluconazole

The interactions of the *C. leptophloeos* B-type dimeric procyanidin (BDP) with synthetic antifungal fluconazole (64–0.06 μg/mL) were investigated using the combination method known as “Checkerboard” in 96-well microtiter plates with RPMI 1640 medium. The dilutions of fluconazole were prepared in order to obtain four times the final concentration in the well, with two other dilutions performed on the microplate itself. The dilutions of BDP were prepared in conical tubes for subsequent application in the microplate, in the concentration range of 2,500–1 μg/mL. For the two-dimensional preparation of the microplates (fluconazole vs. BDP), an amount of 50 μl of each fluconazole concentration was added to columns 1–10. Subsequently, 50 μl of the dilutions of BDP were added to lines A to G. Line H and column 11 contained only fluconazole and BDP, respectively. Column 12 was assigned to wells without drug (growth control) and wells with only the culture medium (sterility control). The fractional inhibitory concentration index (FICI) was used for interpreting the results. The FICI was calculated based on the following equation: FICI = FIC A + FIC B; where, FIC A is the ratio of the combination MIC to the MIC of substance A alone, and FIC B is the ratio of the MIC of the combination to the MIC of substance B alone. The interaction was defined as synergistic, indifferent, and antagonistic if the FICIs were0.5, 0.5–4.0, and 4, respectively (Aslani et al., 2018).

### Statistical Analysis

The data obtained were expressed as mean ± SD. Differences in statistical analyses were evaluated by One-way analysis of variance (ANOVA) with Tukey’s test and the Spearman coefficient was used to assess the correlation between the different techniques employed for quantifying the biofilm formed. Both methods were executed using GraphPad Prism version 5.0 (GraphPad software, San Diego, CA, United States), with statistical significance of ^∗∗∗^*p* < 0.001, ^∗∗^*p* < 0.01, and ^∗^*p* < 0.05.

## Results

### Phytochemical Study of *Commiphora leptophloeos*

**FIGURE 1:**
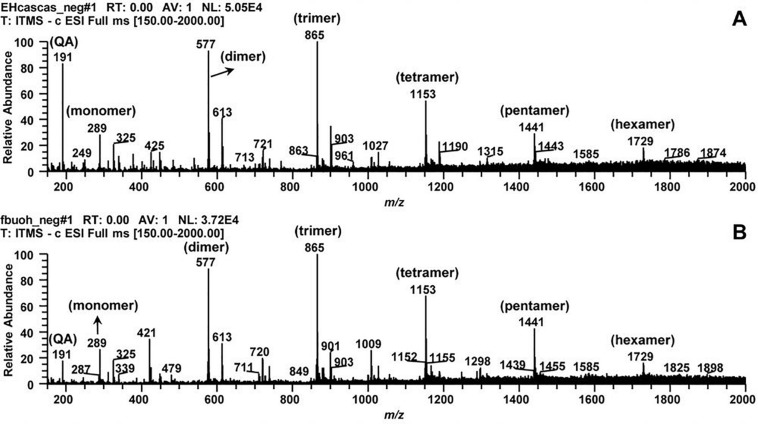
Typical direct flow injection ESI-IT-MS fingerprint spectra obtained in negative ion mode for *Commiphora leptophloeos* hydroethanolic extract in mass scan in the range of *m/z* 210–2000. **(A)** *Commiphora leptophloeos* hydroethanolic extract (HECL). **(B)** *n*-butanol fraction (BF). QA, quinic acid. Monomer, dimer, trimer, tetramer, pentamer, and hexamer of procyanidins.

The phytochemical profile of HECL and BF was initially characterized by mass spectrometry which indicated the presence of quinic acid and oligomeric procyanidins derived exclusively from flavan-3-ol. The second order fragmentation (MS/MS) of the precursor ion at *m/z* 191 led to the identification of quinic acid (QA). The main precursor ions at *m/z* 289, 577, 865, 1,153, 1,441, and 1,729 allowed the identification of the oligomeric series composed of one to six units of flavan-3-ols (monomer, dimer, trimer, tetramer, pentamer, and hexamer, respectively), thus confirming the presence of B-type oligomeric procyanidins (Figures 1A,B and Table 1).

**TABLE 1:** Compounds identified in *Commiphora leptophloeos* hydroethanolic extract (HECL) and n-butanol fraction (BF) by FIA-ESI-IT-MS/MS.

Compound identified	[M-H]^–^ *m/z*	MS/MS *m/z*	References
Quinic acid	191	173, 85	Zhang et al., 2018
Flavan-3-ol (catechin)	289	271, 137	Jiang et al., 2020
B-type dimeric procyanidin	577	451, 425, 289	Jiang et al., 2020
B-type trimeric procyanidin	865	577, 289	Jiang et al., 2020
B-type tetrameric procyanidin	1,153	865, 577, 289	Jiang et al., 2020
B-type pentameric procyanidin	1,441	1,153, 865, 577, 289	Jiang et al., 2020
B-type hexameric procyanidin	1,729	1,441, 1,153, 865, 577, 289	Jiang et al., 2020

**FIGURE 2:**
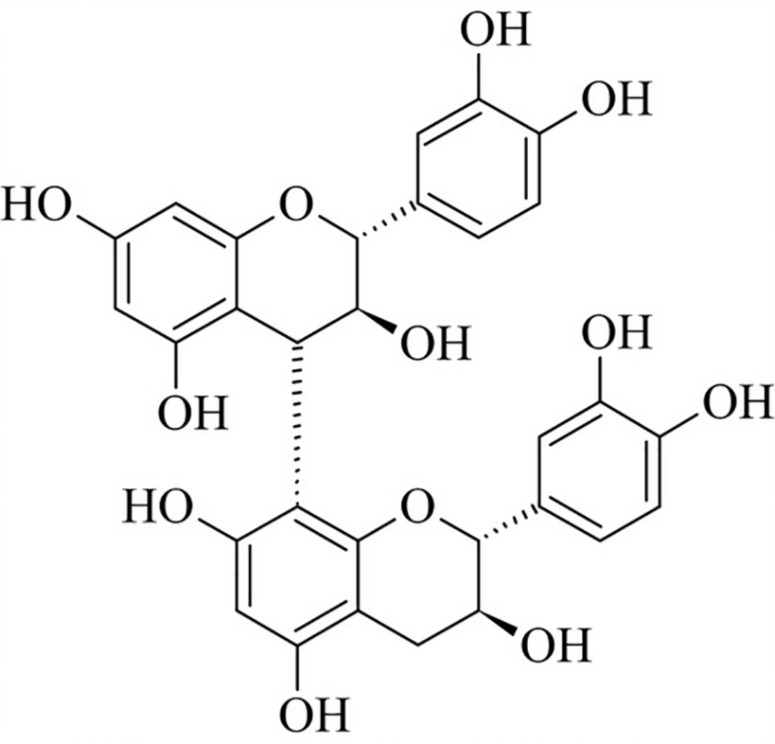
Chemical structure of B-type dimeric procyanidin (BDP).

It is worth noting that, although HECL and BF exhibited the same phytochemical profile, the CTP of BF (383.67 ± 0.02 mg of GAE/g) was higher compared to that of HECL (350.82 ± 0.05 mg of GAE/g). The liquid-liquid extraction helped concentrate the polyphenols in BF, which, through fractioning by CPC, resulted in 25 sub-fractions (SF). These sub-fractions were analyzed by TLC and subsequently by ^1^H NMR and mass spectrometry. The results obtained from the analysis showed that SF-6 was a pure compound (purity > 95% HPLC) identified as B-type dimeric procyanidin (BDP) (Figure 2). The BDP was isolated as a brownish white powder; the result obtained from the analysis of the BDP by TLC showed a single red band after revelation with vanillin sulfuric. The quantity of BDP yielded was 89 mg (1.48%); this was obtained after 8 h of CPC analysis.

BDP analysis by MS spectrometry showed a molecular ion at m/z 577 [M-H]^–^, which was found to be compatible with the molecular formula C_30_H_25_O_12_ (Figure 1 and Table 1). This result is in line with the reports in the literature (Czerwińska et al., 2018). Finally, the chemical structure of BDP was elucidated and confirmed through a comparative analysis of the ^1^H NMR spectra and the data from studies published in the literature (Shoji et al., 2003). The ^1^H NMR spectra of the BDP exhibited methylene proton signals at δH 2.53 and 2.58, which were assigned to the 4-position of the A unit as a (+)-catechin moiety. Next, the proton signals at the 2- and 3-positions of the A unit were assigned to δH 4.96 and 4.16. The interflavanoid bond between units A and B was assigned to the proton signal (δH 4.61) at position 4 of unit B and the proton signal (δH 4.95) at position 2 of unit A (Figure 2 and Supplementary Figure S1). The combination of the results obtained from the UV, MS, and 1H NMR analyses helped determine that the compound isolated from BF was B-type dimeric procyanidin.

**FIGURE 3:**
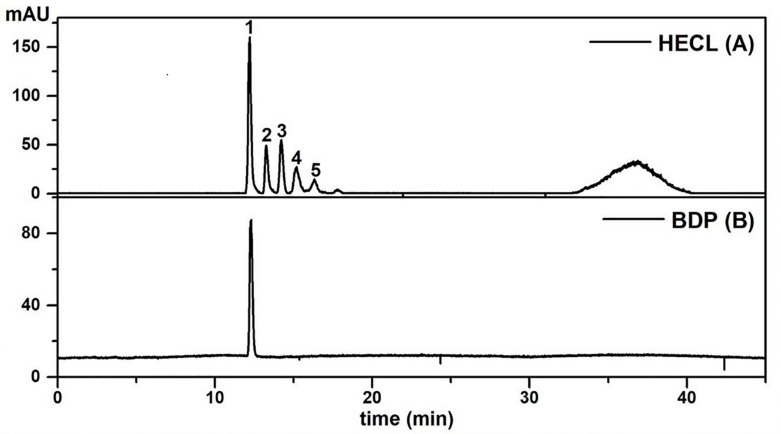
HPLC-ELSD profiles of *Commiphora leptophloeos* hydroethanolic extract and isolated compound employed under the conditions described in the chromatographic section. **(A)** *Commiphora leptophloeos* hydroethanolic extract (HECL). **(B)** B-type dimeric procyanidin (BDP). Numbered chromatographic peaks refer to quantified compounds.

The HPLC-PD/ELSD method (Figure 3) used for rapid simultaneous quantification of the total content of procyanidins in HECL proved to be specific and selective. A comparison of all the peaks of the chromatographic profile of the extract (HECL) and the BDP revealed a single maximum UV absorption band at 280 nm (Supplementary Figure S2), which was found to be typical for flavan-3-ol derivatives (Vidal-Gutiérrez et al., 2020), and spectral similarity indices greater than 0.99 (Tables 2, 3).

**TABLE 2:** Validation parameters of the analytical method for quantifying the content of B-type oligomeric procyanidin in *Commiphora leptophloeos* hydroethanolic extract by HPLC-PAD/ELSD.

Compound	*Rt (min)*	Curve calibration	r	LOD (μ g/mL)	LOQ (μ g/mL)	Precision (%RSD)
**1**	12.39	*y* = 4795.3×− 106	0.9902	21.90	66.38	4.50

*1: BDP; r: coefficient of correlation; Rt, retention time; LOD, detection limit; LOQ, quantitation limit; r, RSD, relative standard deviation; n = 5.*

**TABLE 3:** Peak number, retention time, indices of spectral similarity and content of oligomeric procyanidins in *Commiphora leptophloeos* hydroethanolic extract (HECL) by HPLC-PDA/ELSD.

Peak	*Rt (min)*	Similarity index	Compound content (mg/g of HECL)	Compound name
1	12.39	1.0000	64.16	B-type dimeric procyanidin
2	13.07	0.9992	35.10	B-type trimeric procyanidin
3	13.96	0.9986	38.50	B-type tetrameric procyanidin
4	14.65	0.9978	33.33	B-type pentameric procyanidin
5	15.90	0.9973	27.44	B-type hexameric procyanidin

As shown in Figure 3, the application of the HPLC-PDA/ELSD technique resulted in a separation with a high resolution of the compounds of interest. Peaks 1–5 identified in HECL were quantified using the BDP calibration curves by the external standard method. The BDP calibration curve was linear with a correlation coefficient *r* = 0.9902, while the values obtained for LOD (21.90 μg/mL), LOQ (66.38 μg/mL), and precision (% RSD ≤ 4.50) were found to be satisfactory (Table 2).

### *In vitro* Hemolytic Activity

**FIGURE 4:**
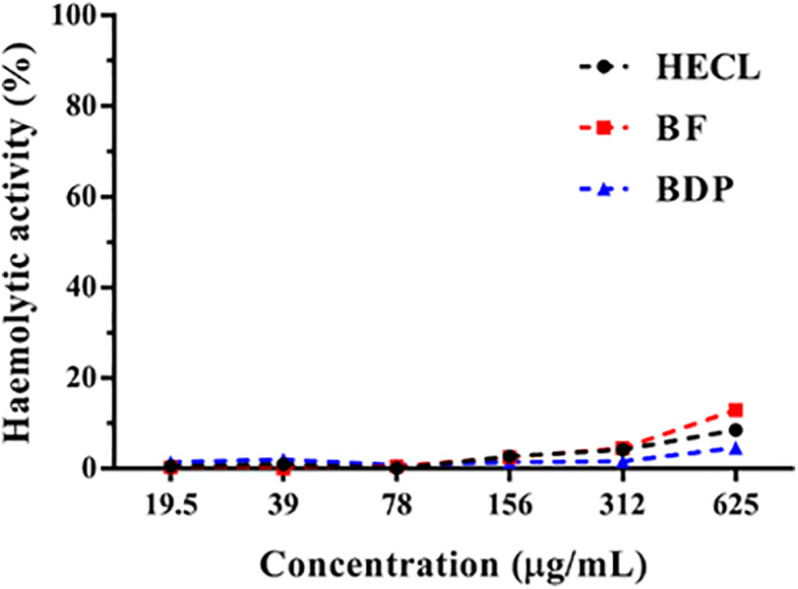
Percentage of hemolytic activity of *Commiphora leptophloeos* hydroethanolic extract (HECL), *n*-butanol fraction (BF), and B-type dimeric procyanidin (BDP).

The cytotoxicity of the HECL, BF, and BDP of *C. leptophloeos* in human erythrocytes was evaluated by testing the hemolytic activity in various concentrations (19.5–625 μg/mL). The HECL, BF, or BDP were not found to be cytotoxic, since they did not present a significant effect on the lysis of human erythrocytes under all the concentrations tested in the study (Figure 4). The hemolytic activity assays served as a basis for determining the concentrations used for assessing antifungal activity.

### Antifungal and Antibiofilm Activities

Possible antifungal activity of the HECL, BF and BDP of *C. leptophloeos* was evaluated against a panel of 14 clinical and reference strains (including fluconazole-resistant strains) of the *Candida* spp. through the determination of MIC and MFC using the broth microdilution test. All the three natural compounds (HECL, BF, and BDP) evaluated here exhibited antifungal activity against all the clinical and reference strains of the *Candida* spp. tested. However, the inhibition concentrations varied among the isolates. The MICs ranged from 156.2 to 2,500 μg/mL for HECL and BDP, while the MICs for BF varied between 312.5 and 2,500 μg/mL. In addition, BDP showed fungicidal activity; this was determined by the absence of CFU growth observation on the SDA plates after incubation, with concentrations ranging from 1250 to 2,500 μg/mL (Table 4).

**TABLE 4:** Minimal inhibitory concentration (MIC) and minimal fungicidal concentration (MFC) of fluconazole (FCZ), *Commiphora leptophloeos* hydroethanolic extract (HECL), *n*-butanol fraction (BF), and B-type dimeric procyanidin (BDP) against *Candida* spp. determined based on the microdilution technique with visual readings of cell turbidity after 24 h/48 h incubation at 37°C in RPMI 1640.

Strains	FCZ (μg/mL)	HECL (μg/mL)	BF (μg/mL)	BDP (μg/mL)	
				
	MIC*	MIC	MIC	MIC	MFC**
*Candida albicans* (ATCC90028)*^*S*^*	0.125	2,500	2,500	625	1,250
*Candida albicans* (LMMM74)*^*S*^*	0.5	1,250	2,500	625	1,250
*Candida albicans* (LMMM92)*^*S*^*	0.5	2,500	2,500	312.5	2,500
*Candida albicans* (LMMM100)*^*S*^*	2.0	1,250	2,500	1,250	2,500
*Candida glabrata* (ATCC 2001)*^*S*^*	0.5	2,500	2,500	1,250	2,500
*Candida glabrata* (LMMM704)*^*S*^*	0.125	2,500	1,250	625	2,500
*Candida krusei* (ATCC 6258)*^*R*^*	≥ 16	625	312.5	156.2	1,250
*Candida krusei* (LMMM249)*^*R*^*	≥ 16	2,500	2,500	1,250	2,500
*Candida parapsilosis* (ATCC 22019)*^*S*^*	0.5	2,500	2,500	312.5	2,500
*Candida parapsilosis* (LMMM85)*^*S*^*	2.0	2,500	1,250	625	2,500
*Candida parapsilosis* (LMMM83)*^*R*^*	≥ 16	156.2	625	312.5	2,500
*Candida tropicalis* (ATCC 13803)*^*R*^*	≥ 16	2,500	2,500	1,250	2,500
*Candida tropicalis* (LMMM195)*^*S*^*	2.0	2,500	312.5	1,250	2,500
*Candida tropicalis* (LMMM447)*^*S*^*	2.0	2,500	312.5	312.5	2,500

The assays were performed in triplicate. ^*R*^fluconazole-resistant strains (≥ 8 μg/mL most of Candida spp.; C. glabrata ≥ 64 μg/mL; C. krusei intrinsically resistant); ^*S*^fluconazole-susceptible or SDD strains (≤16 μg/mL); MIC*, minimal inhibitory concentration; MFC**, minimal fungicidal concentration.

**FIGURE 5:**
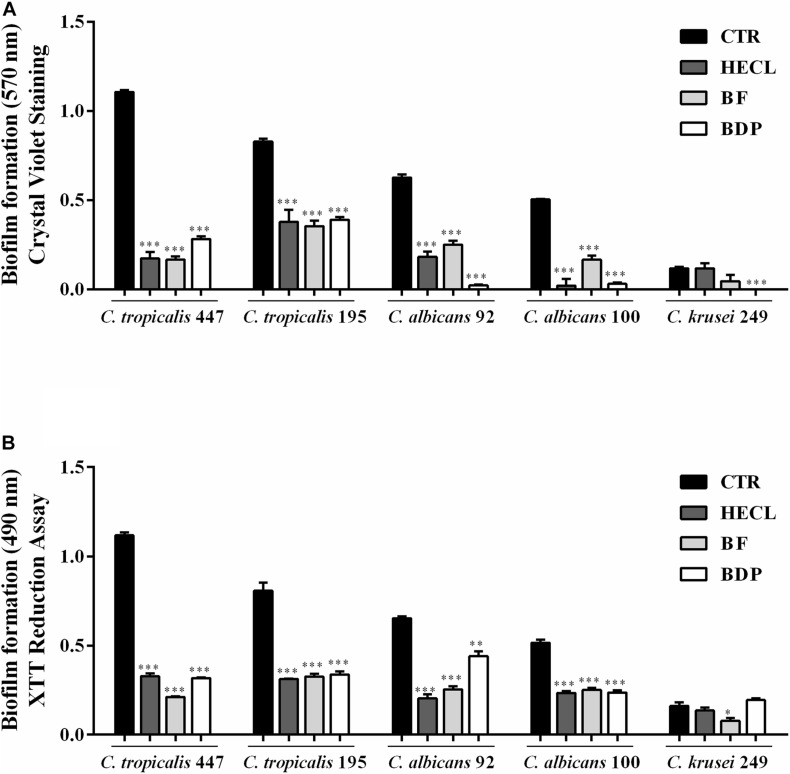
Effect of *Commiphora leptophloeos* hydroethanolic extract (HECL), *n*-butanol fraction (BF), and B-type dimeric procyanidin (BDP) on biofilm of *Candida tropicalis* LMMM447, *C. tropicalis* LMMM195, *C. albicans* LMMM92, *C. albicans* LMMM100, and *C. krusei* LMMM249 clinical isolates. **(A)** Crystal violet and **(B)** XTT assay. Values are expressed as mean ± SD, *n* = 5. ^∗∗∗^*p* < 0.001 compared with control (CTR, medium + yeast). HECL, *Commiphora leptophloeos* hydroethanolic extract (1,250 μg/mL); BF, *n*-butanol fraction (625 μg/mL), and BDP, B-type dimeric procyanidin (625 μg/mL).

The antibiofilm activity of HECL, BF, and BDP was evaluated against five clinical isolates of *Candida* spp. obtained from blood cultures and vulvovaginal secretion using two different quantification methodologies: crystal violet staining and XTT-reduction. All the clinical isolates selected were previously considered biofilm producers. *C. tropicalis* (LMMM447 and LMMM195) and *C. albicans* LMMM92 were considered strong biofilm-producing strains. *C. albicans* LMMM100 was classified as a moderate producer of biofilm, and *C. krusei* LMMM249 was considered a weak biofilm-producing strain, based on the study conducted by Stepanović et al. (2007). It is worth noting that the concentration of 625 μg/mL was used for testing antibiofilm activity in the presence of BF and BDP, whereas a double of this concentration was chosen (1,250 μg/mL) for testing antibiofilm activity in HECL; this is because, in general, higher MICs were necessary to inhibit *Candida* growth in the presence of the hydroethanolic extract. Biofilm formation was found to be significantly impaired (*p* < 0.001) in *C. tropicalis* LMMM447, *C. tropicalis* LMMM195, *C. albicans* LMMM92, and *C. albicans* LMMM100. The percentage of inhibition ranged from 65 to 100% when the cells were treated with HECL, BF, or BDP for both crystal violet staining and XTT-reduction assays. Interestingly, with regard to *C. krusei* LMMM249 strain, only BDP significantly reduced (*p* < 0.001) biofilm formation when crystal violet staining was used for quantification; furthermore, BF showed a statistically significant reduction of biofilm formation when XTT was used for quantifying the metabolic activity of sessile cells. BF and BDP showed a relatively greater antibiofilm potential compared to HECL. Similarly, BDP was able to significantly inhibit (*p* < 0.001) biofilm formation for most of the clinical strains tested, with a percentage of inhibition up to 100% (Figure 5). It is worth pointing out that a positive correlation was found between the two methodologies used for quantifying the formation of biofilm (*r* = 0.8783).

### Analysis of the Synergistic Action of B-Type Dimeric Procyanidin (BDP) From *Commiphora leptophloeos* With Fluconazole

Considering the results obtained from the antifungal activity assay, the combined action of BDP with fluconazole was investigated for the biofilm producing strains. Table 5 shows the results obtained from this analysis. As can be observed, among the strains analyzed, the combination of the two substances was shown to be synergistic for the following strains: *C. albicans* (LMMM 92), *C. albicans* (LMMM100), and *C. tropicalis* (LMMM195). In contrast, with regard to *C. krusei* (LMMM249) and *C. tropicalis* (LMMM447), there was an indifferent effect (without interaction) relative to the combined action of the substances under test; surprisingly though, the values obtained were close to 0.5, and this pointed to a trend of synergistic action even for these strains. The synergistic effect between fluconazole and BDP helped reduce the MIC of this natural product by 4× for *C. albicans* (LMMM100), 8× for *C. albicans* (LMMM92), and 16× for *C. tropicalis* (LMMM447). No antagonism was observed in the combination test for any of the strains investigated.

**TABLE 5:** Synergistic effects of B-type dimeric procyanidin (BDP) from *Commiphora leptophloeos* and fluconazole (FCZ) on *Candida* spp.

Strains		MIC		
	
	BDP (μg/mL)	FCZ (μg/mL)	FICI	Effect
*Candida albicans* (LMMM 92)	2,500	2.0	0.37	Synergistic (8×)
*Candida albicans* (LMMM100)	2,500	2.0	0.50	Synergistic (4×)
*Candida krusei* (LMMM249)	1,250	16.0	0.56	No interaction
*Candida tropicalis* (LMMM195)	1,250	2.0	0.31	Synergistic (16×)
*Candida tropicalis* (LMMM447)	1,250	1.0	0.52	No interaction

*The minimal inhibitory concentration (MICs) values of BDP and fluconazole were obtained in the test, along with the fractional inhibitory concentration index (FICI) and the effect resulting from the interaction of the substances. Values obtained using the checkerboard technique after incubation at 37°C for 48 h.*

## Discussion

### Phytochemical Study of *Commiphora leptophloeos*

Few studies have reported the presence of condensed tannins in species of the *Commiphora* genus. Terpenoids are specialized metabolites which have been widely associated with the genus (Shen et al., 2012; Da Silva et al., 2015). Phytochemical screening studies published in the literature have indicated the presence of tannins in different *C. leptophloeos* stem bark extracts (Trentin et al., 2011; Chaves et al., 2016; Lima et al., 2017). However, the results have not been well understood particularly with respect to the class of tannins, and only few studies have managed to perform a complete phytochemical characterization of these compounds (Trentin et al., 2013; Pereira et al., 2017; Da Silva et al., 2019). To date, quantitative phytochemical data on the species remain scarce. Bearing that in mind, the present study sought to conduct a complete qualitative and quantitative phytochemical characterization of the hydroethanolic extract of *C. leptophloeos* stem bark and investigate the cytotoxic properties, as well as antifungal and antibiofilm activities, of the extract against the strains of *Candida* spp.

The results obtained from our qualitative phytochemical characterization of HECL and BF by MS indicated the presence of quinic acid and six B-type oligomeric procyanidins composed of up to six units of flavan-3-ols. The fragmentation of the precursor ion at *m/z* 191 generated the ions at *m/z* 173 [M-18-H]^–^ and *m/z* 75 [M -116-H] ^–^. This fragmentation pattern, which is in agreement with reports published in the literature, implies the presence of quinic acid (Zhang et al., 2018). The precursor ion at *m/z* 289 was attributed to the deprotonated molecule of flavan-3-ol (catechin), whose fragmentation generated the ions at *m/z* 271 [M-18-H]^–^ and *m/z* 137 [M-152-H]^–^ (Jiang et al., 2020).

The precursor ion at *m/z* 577 indicated the presence of the deprotonated B-type dimeric procyanidin molecule. The fragmentation of this ion produced the ion at *m/z* 451 [M-126-H]^–^, formed by the fragmentation of the heterocyclic ring fission with the elimination of the upper ring A, which indicated the presence of two hydroxyl groups in this ring. The product ion at *m/z* 425 [M-152-H]^–^ was generated by cleavage via Retro Diels-Alder with the elimination of ring B, and indicated the presence of two hydroxyl groups in this ring. Finally, the product ion at *m/z* 289 [M-288-H]^–^ was formed by cleavage via methyl quinone (Czerwińska et al., 2018; Jiang et al., 2020). Thus, two units (upper and lower) of this dimer were identified as flavan-3-ols; as such, the main ions at *m/z* 865, 1,153, 1,441, and 1,729 were found to correspond to the deprotonated molecules of the trimer, tetramer, pentamer, and hexamer of flavan-3-ols, respectively. This outcome was attributed to the consecutive −288 Da losses that occurred through the methyl quinone cleavage pathway, suggesting the presence of B-type oligomeric procyanidins composed of one to six flavan-3-ols units (Jiang et al., 2020; Table 1).

Previous phytochemical studies have reported the presence of phenolic acids, such as gallic acid, chlorogenic acid, protocatequic acid, and A- and B-type polymeric proanthocyanidins, in *C. leptophloeos* extract (Trentin et al., 2013; Pereira et al., 2017; Da Silva et al., 2019). Compared to the previously reported studies in the literature, the distinguishing feature of the phytochemicals found in our technique may possibly be related to the nature of the extractive solution. The extraction of polyphenols depends on the polarity of the solvent due to the greater affinity of these compounds for polar solvents (Tanaka et al., 2018). Thus, the hydroethanolic solvent seemed to have a more adequate polarity to extract greater amounts of phenolic compounds (without interference from other compounds) than the other solvents used previously, such as cyclohexane, chloroform, ethyl acetate, methanol, and water (Pereira et al., 2017). Furthermore, the high solar radiation which characterizes the climate conditions of the Brazilian caatinga biome clearly contributes to great biosynthesis diversity and increases in the content of phenolic compounds present in the plant species (Biasi-Garbin et al., 2016).

Another interesting point worth mentioning is that the present study obtained oligomeric procyanidins-rich fraction with a high polyphenol content which was subjected to fractionation by CPC. The separation method conducted by CPC was found to be efficient, especially for polar substances that are often difficult to separate by classic chromatographic methods (Rohr et al., 2000). The CPC fractionation process, in a single step, enabled us to isolate 89 mg of BDP with high purity, in a few hours of analysis.

BDP belongs to an important class of condensed tannins which has been investigated recently due to its potential health-promoting effects, including antiviral, antibacterial, antileishmanial, anti-inflammatory, and antioxidant effects (Tanaka et al., 2018; Tocci et al., 2018).

Among the novelties of the present work is the fact that it is the first study that has employed the CPC technique for the analysis of *C. leptophloeos*. Trentin et al. (2013) have previously conducted a study which involved the analysis of a polymeric proanthocyanidins-rich fraction from *C. leptophloeos* aqueous extract using classical column chromatography, where the authors were able to identify the constituents of the fraction by MALDI-TOF-MS, but did not obtain pure compounds. Another interesting work that deserves mentioning was the study conducted by Pereira et al. (2017) which isolated a lignan, identified as hinokinin, from cyclohexanic extracts through a tedious fractionation process by flash chromatography using a silica gel column. Considering the scarcity of studies in the literature, the technique adopted in the present study unfolds new possibilities for the application of CPC toward a single-step isolation of bioactive oligomeric procyanidins from *C. leptophloeos*; the technique could also be applied for the characterization of other plant extracts.

To aid the quality control analysis of *C. leptophloeos*, the present study successfully developed an analytical method by HPLC-PDA/ELSD which allowed the simultaneous quantification of five B-type oligomeric procyanidins with good linearity, specificity, precision, and selectivity response and with satisfactory values of LOD and LOQ. Based on the results obtained, BDP was identified as the major substance in HECL, since it is in greater quantity than other substances. In this sense, BDP can be suitably employed as a specific analytical marker for the extract of *C. leptophloeos*. This newly developed HPLC-PDA/ELSD method could potentially be applied for the quantification and authentication of other matrices of natural origin.

### *In vitro* Hemolytic Activity

Considering the adverse effects associated with the prolonged use of antifungal drugs and the fact that these effects can often overlap with the therapeutic effects, the search for bioactive substances with minimal toxicity is of great importance in the development of new antifungal agents (Fuentefria et al., 2018). In this context, it is necessary to assess the toxicity of new natural products with potential antifungal activity in order to have a better understanding of any potentially harmful adverse effects associated with the prolonged use of these products (Campoy and Adrio, 2017).

Based on the results obtained from the hemolytic activity analysis, the HECL, BF, and BDP were found to be safe in biologically active concentrations, since they did not cause significant cytotoxic effects in human erythrocytes. The non-cytotoxic effects observed in this study corroborate with the results obtained by Pereira et al. (2017), in which various *C. leptophloeos* extracts (cyclohexanic, chloroformic, ethyl acetate, methanolic, and aqueous) were found to be safe in the hemolytic activity test. Hexanic and methanolic extracts of *C. leptophloeos* have also been found to exhibit low cytotoxicity against HEp-2 and NCI-H292 tumor cells (Melo et al., 2017). Taking the findings of these studies into account, one can conclude that the low cytotoxicity observed for *C. leptophloeos* may be related, at least in part, to the presence of tannins (Melo et al., 2017; Macedo et al., 2018). In fact, the toxicity of tannins are considerably lower compared to other secondary metabolites with antifungal potential such as alkaloids and terpenoids (Tanaka et al., 2018).

### Antifungal and Antibiofilm Activities

Few studies published in the literature have shed light on the antifungal activity of *C. leptophloeos*, and most of these studies have confined their attention solely to the antibacterial activity of *C. leptophloeos* (Trentin et al., 2013; Pereira et al., 2017; Da Silva et al., 2019). Pereira et al. (2017) recently described the antifungal activity of various *C. leptophloeos* extracts (cyclohexanic, chloroformic, ethyl acetate, methanolic, and aqueous) against reference strains of *C. albicans* and *Aspergillus* sp. Based on the findings of their study, the extracts exerted antifungal activity only when the concentrations were very high, with MIC values ranging from 6.25 to 12.5 mg/mL for *C. albicans*, and from 3.12 to 12.5 mg/mL for *Aspergillus* sp. The concentrations reported by Pereira et al. (2017) were up to 10 times higher than the concentrations employed in our present study for HECL, where MIC values ranged from 312.5 to 2,500 μg/mL for *C. albicans* (Table 4). Similarly, the polyphenols content of HECL in our study was approximately 10 times higher than that of the extracts reported by Pereira et al. (2017). This can be attributed to the fact that the polarity of the extracting solvents directly influences the chemical constitution and the content of secondary metabolites in the extracts, and this consequently affects the antifungal activity (Rohr et al., 2000). Thus, our conclusion is that polyphenols, especially oligomeric procyanidins, are responsible for the stronger antifungal activity observed in the present study.

Our results corroborate with those of Ming et al. (2002) who showed that B-type dimeric and trimeric procyanidins presented antifungal activity against *C. albicans* (MIC = 150 μg/mL). Since these procyanidins have been identified in satisfactory amounts in HECL, we share the view that BDP can act alone or in synergy with other oligomeric procyanidins, such as B-type trimeric procyanidin, to inhibit the growth of *C. albicans* strains or cause their cell death. Furthermore, BDP has been reported to exhibit antibacterial activity against *Staphylococcus aureus* (Ming et al., 2002).

Biofilm is one of the most prevalent forms of growth in nature, defined as a microbial community embedded in an extracellular matrix and adhered to surfaces. Hence, microorganisms in biofilm are more resistant to antimicrobial agents than planktonic cells (Sherry et al., 2017). HECL, BF, and DBP exhibited more than a 65% inhibition rate of biofilm formation for most of the clinical isolates of *Candida* spp. investigated in this study. However, less effective antibiofilm activity was observed for HECL and BF against *C. krusei*. This result may be partially attributed to the fact that the strain tested is a low biofilm producer; apart from that, procyanidins are natural reddish pigments of the anthocyanidin class (Rohr et al., 2000). Although several washing steps are performed before biofilm staining, the pigments might have influenced the optical density (O.D) readings, particularly in this naturally low biofilm producer strain. In addition, despite the intrinsic resistance of *C. krusei* to fluconazole, new functionalized analogs from the fluconazole triazole ring have demonstrated efficacy against fluconazole-resistant isolates of *C. krusei* (Motahari et al., 2018; Mahmoudi et al., 2019).

The most remarkable finding of our study has to do with the effect of BDP in relation to HECL and BF. Based on the results obtained, BDP significantly inhibited biofilm formation in most of the clinical isolates tested; in fact, it exhibited inhibition percentage of up to 100%. Clearly, this points to the importance of obtaining new compounds for the identification of potential antifungal targets.

This result was confirmed through the application of two different methodologies for quantifying biofilm production (crystal violet staining and XTT-reduction assay). It is worth mentioning that these techniques are complementary to one another in the sense that while crystal violet stains biofilm biomass (including the exopolymeric matrix), XTT reduction is related to biofilm metabolic activity. Thus, it is likely that BDP causes cell death and contributes toward the reduction of a well-structured biofilm (Melo et al., 2011). Furthermore, it has been reported that aqueous and ethanolic extracts of *C. leptophloeos* prevented biofilm formation caused by *Staphylococcus* spp. and *Pseudomonas aeruginosa* through bacteriostatic properties (Trentin et al., 2011, 2013; Da Silva et al., 2019).

Although it is evident that oligomeric procyanidins are capable of impairing the full expression of the virulence factors of *Candida* spp., such as biofilm formation, the possible mechanism of action has not yet been fully elucidated. The literature shows that procyanidins are able to reduce the secretion of proteinases, due to their ability to complex with proteins, and can inhibit phospholipase activity (Macáková et al., 2014; Rauf et al., 2019). Tannins have also been shown to rupture the cell wall and plasma membrane, as well as inhibit 1,3-β-D-glucan synthase (PbFKS1) transcript accumulation, which is involved in cell wall synthesis (Zambuzzi-Carvalho et al., 2013; Trolezi et al., 2017). Thus, the antifungal activity of *C. leptophloeos* observed in this study may be related to the reduction of the production of hydrolytic enzymes, biofilm formation, and/or damage of cell wall and plasma membrane (primary cell targets) of *Candida* cells.

The simultaneous use of antimicrobial agents can maximize the chances of success in the therapeutic process, thus improving the spectrum of action, optimizing the time of intervals and treatment doses, and even reducing the concentration of the antifungal product employed (Herrera et al., 2010; De Oliveira et al., 2014; Salazar-Aranda et al., 2015; Li et al., 2017). The synergistic action of BDP with fluconazole observed for most of the strains tested may be considered an interesting alternative for the treatment of fungal infections. The enhanced effect of fluconazole (which targets 14α-demethylase–an intermediate molecule of ergosterol biosynthesis) combined with BDP may possibly be related to the affinity of procyanidins for ergosterol, since they are able to bind to membrane ergosterol, forming a tannin-ergosterol complex and, consequently, reducing the amount of ergosterol in *Candida* cells (Carvalho et al., 2018).

Finally, it is worth pointing out that only *in vitro* tests were used in the present study. Further pre-clinical *in vivo* and clinical studies ought to be carried out in order to obtain a better understanding of the mechanisms of action of *C. leptophloeos* and to validate its efficacy and safety for the treatment of fungal infections.

## Conclusion

The present study reported the presence of B-type oligomeric procyanidins in the stem bark of *C. leptophloeos*, as well as the antifungal and antibiofilm activities of the plant extract against *Candida* spp. This is the first report of its kind in the literature. BDP, which was isolated for the first time in *C. leptophloeos*, showed higher anti-*Candida* activity compared to HELC and FB, with lower MIC and MFC values, as well as greater capacity to reduce biofilm formation. The study showed that the plant extract, *n*-butanol fraction, and the isolated compounds may be used as an alternative complementary mechanism for the treatment of fungal infections, specifically in fluconazole resistant strains and multidrug resistant *Candida* spp. The results of the study showed that free B-type dimeric procyanidin clearly exhibits fungicidal activity; furthermore, the combined application of BDP with fluconazole leads to the enhancement of the antifungal effect via synergistic action. The study also reported the development and application of a HPLC-PDA/ELSD method for direct and simultaneous detection and quantification of five B-type oligomeric procyanidins in the extract of *C. leptophloeos*. Based on the findings of the study, the HPLC-PDA/ELSD method can be applied as an important tool for the quality control of raw materials and/or herbal medicine, given that *C. leptophloeos* is widely used as a medicinal plant in Brazil. BDP was found to be responsible, at least in part, for the pharmacological effect observed in *C. leptophloeos* and can be employed as an analytical marker for the plant species. The findings of the study confirm the antifungal properties and low cytotoxic effects of *C. leptophloeos*, thus providing scientific support for its use in folk medicine toward the treatment of fungal infections.

## Data Availability Statement

The original contributions presented in the study are included in the article/Supplementary Material, further inquiries can be directed to the corresponding author/s.

## Author Contributions

RD-M was responsible for conceptualization, methodology, investigation, design of the experiments, and the writing of the original draft. AZ and WV conducted the qualitative and quantitative phytochemical analyses. LS performed antifungal and antibiofilm experiments. JF, SM, and RG conducted isolation and structural elucidation experiments. BA-C and MF-P performed the cytotoxicity tests. MT-R was responsible for the processing of the statistical data. TA was responsible for collecting, drying, and the botanical identification of the plant material. GC was responsible for conceptualization, supervision, writing review of the manuscript. SZ was responsible for conceptualization, methodology, investigation, writing review, supervision, project administration, and funding acquisition. All authors contributed equally toward the elaboration of the manuscript and have given their approval of the final version.

## Conflict of Interest

The authors declare that the research was conducted in the absence of any commercial or financial relationships that could be construed as a potential conflict of interest.
