# Supplementary material for: Antifungal and Antibiofilm Activities of B-Type Oligomeric Procyanidins From Commiphora leptophloeos Used Alone or in Combination With Fluconazole Against Candida spp
Source: Front Microbiol. 2021 Feb 22;12:613155. doi: 10.3389/fmicb.2021.613155 (PMC7937886; doi:10.3389/fmicb.2021.613155)
Supplement: Supplementary file 1 [file Data_Sheet_1.docx]

Supplementary Material

# Supplementary Figures

**Supplementary Figure 1.** ^1^H NMR spectrum of B-type dimeric procyanidin (MeOD, 400 MHz).


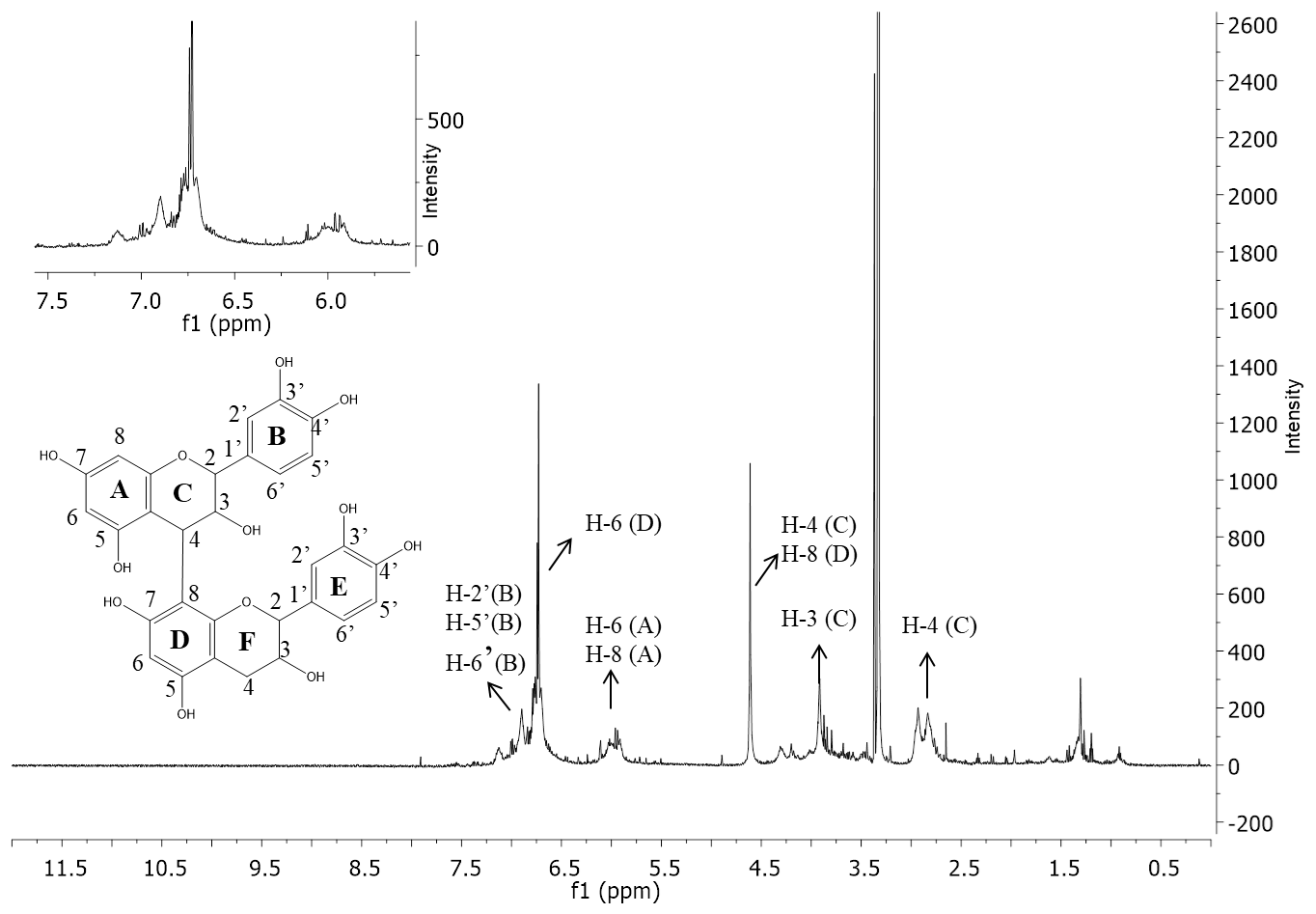


**Supplementary Figure 2.** The ultraviolet absorption spectrum of peaks **1** to **5** of the hydroethanolic extract of *C. leptophloeos* (HECL) by HPLC-PDA/ELSD (UV max = 280 nm).

**
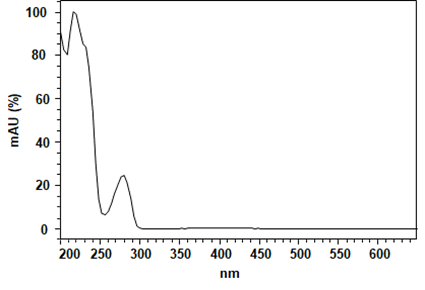
**
